# Supplementary material for: Validity and Absolute Reliability of the Cobb Angle in Idiopathic Scoliosis with TraumaMeter Software
Source: Int J Environ Res Public Health. 2022 Apr 12;19(8):4655. doi: 10.3390/ijerph19084655 (PMC9027061; doi:10.3390/ijerph19084655)
Supplement: Supplementary file 1 [file ijerph-19-04655-s001.zip › TABLE S1a_TRAUMAMETER & COBB_IJERPH.pdf]

	E11	E12	E13	E21	E22	E23	E31	E32	E33	E41	E42	E43	N11	N12	N13	N21	N22	N23	N31	N32	N33	N41	N42	N43
**1a**	12.4	12.0	12.8	13.1	13.3	13.4	12.2	12.3	12.8	11.1	11.8	13.4	9.3	11.4	10.9	15.2	13.4	13.9	14.7	12.4	13.5	12.3	11.5	12.7
**2a**	15.8	14.2	15.3	18.0	17.1	16.9	16.1	17.8	16.7	19.7	19.4	20.0	16.2	16.9	18.5	17.9	16.9	18.4	17.1	16.8	15.7	17.3	15.3	18.1
**3a**	18.9	20.1	20.7	19.1	19.6	19.8	21.5	20.7	20.4	19.6	22.4	20.4	21.5	21.9	18.5	22.4	22.6	20.9	20.4	19.2	20.6	17.3	20.0	18.2
**4a**	30.5	29.9	31.2	32.2	28.9	31.7	36.7	36.6	34.3	33.2	32.3	32.8	32.7	30.5	29.1	32.8	31.3	32.9	34.3	35.8	32.2	29.7	33.5	26.9
**5a**	32.3	28.3	28.8	31.4	30.5	31.1	31.9	32.4	32.0	30.8	34.5	32.9	31.9	32.1	30.5	31.4	30.8	29.7	30.6	31.1	32.1	31.9	33.1	28.8
**6a**	43.1	41.6	42.1	40.2	46.1	45.4	46.2	47.8	47.6	50.3	48.8	51.3	41.3	45.7	49.3	45.4	44.4	41.7	44.4	43.7	44.9	46.7	42.5	42.4
**7a**	24.6	23.7	25.6	24.9	23.9	24.3	24.2	24.6	24.2	20.9	26.1	27.0	25.4	25.8	23.8	24.1	25.3	25.6	24.1	23.8	25.0	24.0	25.6	21.6
**8a**	46.6	44.6	47.4	45.2	44.4	44.9	44.6	44.3	45.2	43.7	46.1	46.1	43.8	45.8	47.1	45.9	47.4	44.3	42.0	43.6	44.8	44.2	47.9	42.0
**9a**	36.2	36.3	37.0	36.1	36.5	36.2	41.1	40.4	40.1	37.0	40.4	42.4	40.9	36.6	36.0	37.4	38.0	40.0	36.6	39.4	38.1	37.6	37.0	25.4
**10a**	19.3	16.5	19.2	20.2	17.9	19.9	18.6	17.3	17.2	18.3	19.9	20.7	18.0	19.8	18.9	19.2	18.0	18.4	18.4	17.1	18.7	18.5	19.1	18.3
**11a**	25.7	26.2	24.5	28.9	28.2	26.3	27.6	27.6	27.2	24.5	26.0	27.1	28.5	26.4	25.9	28.0	31.9	28.1	27.1	27.2	27.0	26.9	28.2	27.4
**12a**	34.6	34.7	35.1	11.5	36.5	37.7	39.7	39.2	38.4	36.2	40.7	39.5	35.3	36.4	37.5	38.2	37.9	37.6	35.0	36.0	37.2	35.5	38.2	38.4
**13a**	54.3	55.9	54.8	55.8	55.4	55.5	55.4	55.4	55.9	57.0	56.8	58.2	55.7	52.4	53.9	57.3	56.3	55.7	55.8	56.9	57.7	57.4	55.7	55.9
**14a**	30.4	29.9	30.9	29.7	29.1	30.2	29.0	29.5	29.2	31.8	30.8	26.7	29.5	29.5	31.1	33.0	31.0	29.0	26.6	28.9	35.4	30.6	30.2	31.4
**15a**	65.0	62.8	66.0	65.9	64.7	67.1	66.8	66.8	65.8	65.3	69.2	67.3	67.2	60.8	68.8	70.4	68.6	71.2	67.5	65.5	65.9	58.6	61.8	66.6
**16a**	20.0	18.7	20.6	19.8	19.1	20.4	18.8	19.4	19.9	25.3	21.0	20.5	25.2	21.0	20.4	21.0	21.3	22.7	23.7	21.3	19.7	19.7	22.1	20.8
**17a**	55.6	55.7	56.1	56.5	57.4	58.9	55.6	55.4	55.1	59.1	56.5	59.3	53.7	56.9	52.3	56.0	56.8	56.6	57.0	56.9	56.4	56.5	59.2	56.6
**18a**	56.5	54.1	54.2	56.3	56.1	54.6	57.7	58.6	57.7	57.7	60.7	60.0	60.7	50.1	53.6	55.5	57.7	56.3	58.3	56.4	55.3	57.6	56.4	59.0
**19a**	35.8	35.9	34.9	38.3	37.3	38.7	37.4	37.7	38.0	37.7	39.9	42.5	36.0	38.3	40.5	39.3	38.4	37.8	35.4	38.1	38.1	38.0	35.9	37.4
**20a**	13.7	13.7	15.1	14.6	16.0	16.6	17.7	17.1	17.4	18.7	18.2	16.9	14.6	14.5	16.4	19.2	16.3	15.9	14.6	15.6	17.5	13.6	14.4	13.3
**21a**	55.5	57.7	54.7	53.9	59.0	53.4	53.0	52.7	52.9	55.6	54.8	56.4	54.7	56.8	57.7	66.7	63.2	64.3	57.4	52.9	54.4	55.8	50.8	51.3
**3b**	14.5	15.0	15.6	18.7	15.8	16.6	17.0	16.4	16.5	16.9	18.6	17.6	13.7	18.6	18.3	16.1	17.4	18.4	16.1	17.1	18.6	19.0	16.2	16.4
**4b**	25.4	23.5	27.3	25.7	26.4	25.0	23.8	23.1	23.4	27.4	26.3	26.1	23.2	24.4	23.8	26.0	27.1	27.1	27.5	24.8	25.4	27.5	24.8	24.6
**6b**	30.6	28.9	30.8	28.9	29.3	31.3	27.2	26.1	26.3	30.1	31.9	33.5	28.5	32.8	36.5	30.0	30.1	29.8	30.2	31.6	31.1	29.6	29.3	28.5
**7b**	16.4	18.7	17.7	14.7	15.5	17.4	16.6	16.8	16.8	15.8	16.4	17.2	17.0	18.6	18.0	17.3	18.3	18.4	15.6	17.8	16.2	17.5	16.6	16.1
**10b**	15.6	15.2	17.7	16.8	17.9	18.6	15.7	15.8	15.6	15.8	16.5	17.7	17.8	16.5	17.4	14.7	17.3	17.6	17.8	16.4	17.1	15.0	17.2	15.9
**11b**	23.7	23.7	23.1	25.1	26.3	25.8	26.1	26.6	26.6	24.5	27.2	26.8	21.7	21.7	21.7	25.0	26.4	27.3	27.0	26.4	26.1	26.5	24.5	23.9
**12b**	33.1	33.3	33.2	7.5	33.6	35.6	33.1	33.8	33.0	34.6	35.2	33.0	33.5	33.9	30.1	32.0	34.8	34.2	32.2	31.3	33.3	32.0	32.7	34.0
**13b**	35.4	35.3	35.9	35.1	35.0	36.8	38.3	37.4	37.5	34.7	36.4	34.6	36.7	35.1	38.0	36.5	36.8	34.2	33.1	34.3	32.3	32.9	38.3	34.4
**14b**	26.4	25.1	26.5	26.6	26.7	28.8	27.7	27.7	27.6	27.2	27.8	28.9	28.6	26.4	27.6	27.1	26.7	27.2	26.3	28.5	26.2	30.6	30.3	29.6
**15b**	50.8	50.5	50.7	46.2	46.1	45.1	49.9	50.2	50.4	44.8	48.7	42.2	48.6	41.7	49.1	49.3	51.8	51.1	50.1	44.9	48.6	44.9	48.8	50.2
**16b**	18.8	18.3	19.4	20.1	18.4	20.2	18.7	18.3	18.9	19.7	19.1	18.3	20.3	20.7	17.1	19.7	20.6	19.6	20.2	15.8	17.2	13.4	17.0	15.9
**17b**	39.2	36.8	37.0	37.6	36.2	37.3	39.3	39.0	38.9	39.5	39.6	40.1	37.5	36.4	35.4	38.8	39.4	39.3	39.3	39.1	33.1	38.1	32.4	33.4
**18b**	40.3	39.3	38.8	38.1	40.0	37.7	36.2	36.8	36.4	41.1	39.1	42.3	32.9	36.5	38.3	38.4	38.2	38.5	35.9	38.1	38.1	37.1	39.8	29.5
**21b**	45.4	47.0	46.8	46.7	48.5	48.4	52.4	52.1	52.4	50.8	49.8	50.6	47.4	47.4	50.7	46.3	48.8	50.2	45.8	49.7	49.7	47.7	46.2	48.1

Table S1a. Cobb angle values (in degrees) of each curve obtained by each observer and in each series of measurements, with the software. Xa is the primary curve, and Xb is the secondary or compensating curve of the RX number X (X1 to X21). The first digit of the column descriptors corresponds to the observer and the second to the measurement series (e.g., E21 is equivalent to the first measurement of the "Expert observer" number 2). E, a measurement obtained by the "Expert observer"; N, the measurement from the "Novel observer".
