# Supplementary material for: Validity and Absolute Reliability of the Cobb Angle in Idiopathic Scoliosis with TraumaMeter Software
Source: Int J Environ Res Public Health. 2022 Apr 12;19(8):4655. doi: 10.3390/ijerph19084655 (PMC9027061; doi:10.3390/ijerph19084655)
Supplement: Supplementary file 1 [file ijerph-19-04655-s001.zip › TABLE S2b_TRAUMAMETER & COBB_IJERPH.pdf]

**1a**			
**2a**			
**3a**	25	25	
**4a**	20		
**5a**			
**6a**	32		
**7a**			
**8a**			
**9a**			
**10a**			
**11a**	33	22	
**12a**			
**13a**			
**14a**	35		
**15a**	57		
**16a**			
**17a**	42		
**18a**			
**19a**	27		
**20a**			
**21a**	75		
**3b**	13		
**4b**			
**6b**			
**7b**	26	24	
**10b**			
**11b**	16		
**12b**	27		
**13b**			
**14b**	22	20	20
**15b**			
**16b**	35		
**17b**			
**18b**	23		
**21b**	39	39	

Table S2b. Removed outliers from the measurements distributions of each scoliotic curve obtained by observers using the manual method. Xa is the primary curve, and Xb is the secondary or compensating curve of the RX number X (X1 to X21).
