# Supplementary material for: Validity and Absolute Reliability of the Cobb Angle in Idiopathic Scoliosis with TraumaMeter Software
Source: Int J Environ Res Public Health. 2022 Apr 12;19(8):4655. doi: 10.3390/ijerph19084655 (PMC9027061; doi:10.3390/ijerph19084655)
Supplement: Supplementary file 1 [file ijerph-19-04655-s001.zip › TABLE S2c_TRAUMAMETER & COBB_IJERPH.pdf]

**SoftE1E2**	4.01			
**SoftE2E3**				
**SoftE1E3**	4.36	3.55		
**SoftN1N2**	4.18	3.73	3.62	
**SoftN2N3**	5.46			
**SoftN1N3**	4.08			
**SoftE**	3.98			
**SoftN**	4.66			
**SoftE1N1**	3.59	3.28		
**SoftE2N2**	4.49	3.89		
**SoftE3N3**	4.39			
**ManuE1E2**				
**ManuE2E3**	5.13			
**ManuE1E3**	5.00			
**ManuN1N2**	7.42	5.63		
**ManuN2N3**				
**ManuN1N3**	6.75	4.75	4.67	4.63
**ManuE**				
**ManuN**	5.95			
**ManuE1N1**	6.75			
**ManuE2N2**				
**ManuE3N3**	6.19	5.81		
**SoftEN**	4.26	3.58		
**ManuEN**	5.55			

Table S2c. Removed outliers from each error distribution. “Soft” refers to measurements obtained with the software, and “Manu” refers to measurements obtained manually. EX refers to the measurements obtained by Expert measurers in measurement round X. NY refers to the measurements obtained by the Novice measurers in measurement run Y. (e.g., ManuE1N1 refers to the distribution of errors between the measurements obtained manually by the group of Experts and the group of Novices in the first measurement run; SoftN refers to the distribution of errors between the measurements obtained with the software by the group of Novices).
